# Porous silicon and silica carriers for delivery of peptide therapeutics

**DOI:** 10.1007/s13346-024-01609-7

**Published:** 2024-05-31

**Authors:** Jiachen Yan, Prakriti Siwakoti, Siuli Shaw, Sudeep Bose, Ganesh Kokil, Tushar Kumeria

**Affiliations:** 1 School of Materials Science and Engineering, The University of New South Wales, Sydney, NSW 2052 Australia; 2 Australian Centre for Nanomedicine, The University of New South Wales, Sydney, NSW 2052 Australia; 3 Centre for Medical Biotechnology, Amity Institute of Biotechnology, Amity University, Noida, Uttar Pradesh 201301 India; 4 Amity Institute of Molecular Medicine and Stem Cell Research, Amity University, Noida, Uttar Pradesh 201301 India; 5 School of Pharmacy, The University of Queensland, Woolloongabba, QLD 4102 Australia

**Keywords:** Porous materials, Drug delivery systems, Nanocarriers, Peptide drugs, Peptide encapsulation

## Abstract

Peptides have gained tremendous popularity as biological therapeutic agents in recent years due to their favourable specificity, diversity of targets, well-established screening methods, ease of production, and lower cost. However, their poor physiological and storage stability, pharmacokinetics, and fast clearance have limited their clinical translation. Novel nanocarrier-based strategies have shown promise in overcoming these issues. In this direction, porous silicon (pSi) and mesoporous silica nanoparticles (MSNs) have been widely explored as potential carriers for the delivery of peptide therapeutics. These materials possess several advantages, including large surface areas, tunable pore sizes, and adjustable pore architectures, which make them attractive carriers for peptide delivery systems. In this review, we cover pSi and MSNs as drug carriers focusing on their use in peptide delivery. The review provides a brief overview of their fabrication, surface modification, and interesting properties that make them ideal peptide drug carriers. The review provides a systematic account of various studies that have utilised these unique porous carriers for peptide delivery describing significant in vitro and in vivo results. We have also provided a critical comparison of the two carriers in terms of their physicochemical properties and short-term and long-term biocompatibility. Lastly, we have concluded the review with our opinion of this field and identified key areas for future research for clinical translation of pSi and MSN-based peptide therapeutic formulations.

**Graphical abstract:**

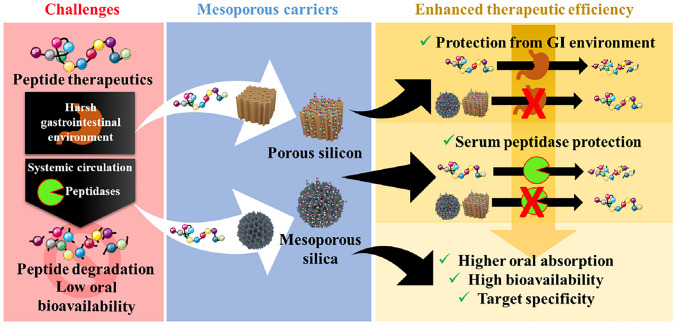

## Introduction

Peptide therapeutics are a class of drugs composed of short chains of amino acids. They typically consist of fewer than 50 amino acid residues linked together through amide bonds between the amino (-NH_2_) and carboxylic (-COOH) groups of adjacent amino acids [1]. As signalling molecules, peptides can selectively bind to receptors with specific surface structures, triggering intracellular effects [2]. Depending on the purpose, peptide therapeutics have been applied to treat different diseases, such as cancer [3], cardiovascular diseases [4, 5], infectious diseases [6], pain management [7], neurological [8, 9], and metabolic disorders [10]. Apart from therapeutic applications, peptide-based probes are also immensely popularly used in bioimaging systems when assembled along with imaging components, for better observing and understanding processes like metabolism, gene expression, receptor binding, and biochemical pathways in real-time [11, 12]. This topic has been comprehensively covered in recent review articles from Wang and Hu [11] as well as Honggang Cui’s group [12].

Peptide therapeutics have become increasingly popular due to their outstanding characteristics, including their high specificity, low toxicity, and ability to target specific cellular pathways [13, 14]. Peptides are typically metabolised and excreted from the body relatively quickly, reducing the risk of toxicity or accumulation in the body [15]. This can be particularly important in the treatment of chronic diseases, where patients may need to take medication for an extended period. Like antibody-based biologics, therapeutic peptides bind target cells with high affinity and specificity [1], minimising repeated dosing and off-target effects. One potential disadvantage of peptide therapeutics is that they can be immunogenic, meaning they can trigger an immune response in the body [16]. This can lead to the development of antibodies against the peptide therapeutics, which can reduce the effectiveness of the treatment or cause adverse reactions [16, 17]. In recent years, the issue of immunogenicity of peptide therapeutics has been addressed using surface modification, such as PEGylation [18], glycosylation [19], and lipidation [20], which also improve their safety and tolerance [21]. Compared to proteins, peptides have shorter amino acid chains and simpler structures, which makes them more effective in penetrating cell membranes [22]. Additionally, peptides can be easily synthesised using solid-phase peptide synthesis techniques, which allows the production of large quantities of peptides in a relatively short amount of time [23]. However, the route of administration plays a key role in determining the efficacy and safety of the peptides in vivo. For example, when delivered through oral administration, peptides are highly prone to degradation by proteolytic enzymes and suffer from weak mucus layer penetration, which is responsible for their low systemic absorption (i.e. bioavailability) [24]. Systemic administration through the intravenous [14] route provides high bioavailability but is limited by the short circulation half-life and rapid bioavailability decrease due to fast kidney and liver clearance [25]. Topical and transdermal delivery of peptides holds potential for providing local relief for the patients, but is severely limited by the permeation of macromolecular peptides and degradation by enzymes in the skin [26]. Therefore, it is necessary to utilise the appropriate route of administration and delivery system to maximise the efficacy of the peptide therapeutics to shield them against degradation and enable controlled release. Peptide delivery still requires further investigation and advancements, although many have been confirmed to be feasible by the United States Food and Drug Administration (US-FDA) [27] and applied in clinic.

A number of nanocarriers have been explored for encapsulating peptide therapeutics to improve their stability [28–30] and bioavailability [31–33] in in vivo delivery. Among the prevalent nanocarriers, systems based on lipids and polymers are notable. For a comprehensive understanding, refer to the recent reviews by Bernkop-Schnürch et al. and Lozano et al. on use of lipid-based carriers for peptide delivery [34, 35], and the review by Sung and Kim concerning polymer-based nanocarriers [36]. However, a majority of studies employing lipids and polymer-based systems for this purpose often suffer from poor peptide encapsulation rate, leaching, and inability to incorporate larger peptides. In recent years, mesoporous nanocarriers such as porous silicon (pSi) and mesoporous silica nanoparticles (MSNs) [37] have garnered considerable attention as delivery systems to formulate peptide therapeutics. Although other porous materials like MoFs, also offer a porous structure, both pSi and MSNs offer tuneable porous structures with pore sizes ranging from 2–50 nm, which is better suited for macromolecular payloads like peptides [38, 39]. These porous structures are able to hold peptides encapsulated with minimal premature release during in vivo transportation and circulation [40]. In addition, these nanoparticles demonstrated high thermal and chemical stability as well as [41], thus can be suitable for deployment in a variety of therapeutic peptides in in vivo environments. Additionally, the targeting capacity and biocompatibility of pSi and MSNs templates can be further improved by surface modification after loading therapeutic [38, 39] while a number of studies have utilised surface-grafted peptides as targeting moieties.

**Fig. 1:**
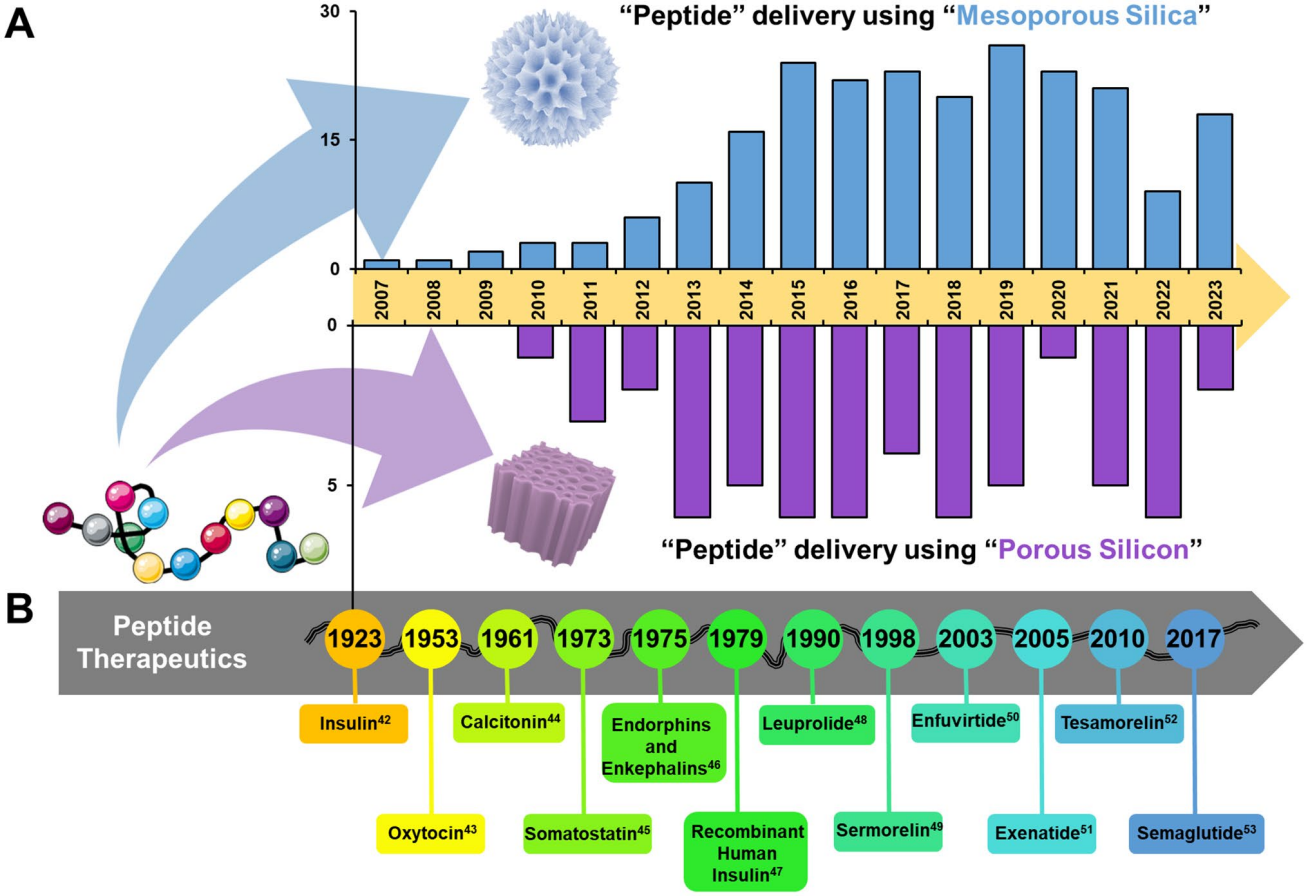
Advancement in peptide delivery using pSi and MSN carriers. **A** The number of peer-reviewed studies conducted annually for both nanocarriers from 2007 to 2023. The data presented in the bar-graph is from Scopus, collected in Nov 2023. The keywords used for MSN and pSi are listed in the figure. **B** Key milestone in development and the United States Food and Drug Administration (US-FDA) approval of peptide therapeutic [42–53]

This review summarises the current research on the use of pSi and MSNs as nanocarriers for the delivery of peptide therapeutics. Figure 1A provides a glimpse of the progress in use of these carriers for delivery of peptides. Specifically, the focus is on the properties of pSi and MSNs that make these carriers ideal for drug delivery, in particular peptide therapeutics. Subsequently, a detailed account of the recent literature on the use of these two porous carriers for peptide delivery through systemic, oral, and topical routes is provided. The review also provides a critical comparison between the pSi and MSNs as suitable carriers for macromolecular payload delivery and medical application. Finally, the challenges associated with pSi- and MSNs-based peptide delivery systems are discussed, along with an assessment of the potential clinical opportunities for their use as therapeutic peptide delivery systems.

## List of peptide-based drugs approved by US-FDA

The discovery and development of macromolecular therapeutics has come a long way with a number of biologics currently approved for human use. As of now, over 300 biologics are approved by the US-FDA. Of all the biologic drugs sanctioned by the US-FDA, close to 80 are derived from peptides, signifying a promising trajectory for clinical application. Key milestones in the development of peptide therapeutics since the discovery of insulin in 1923 are shown in Fig. 1B. Besides, a list of peptide drugs that have received US-FDA approval since 2018 is provided in Table 1. The table presents information about their active constituents, therapeutic indications, targeted receptors, and administration routes. Notably, certain peptides can function directly as therapeutic drugs, such as Setmelanotide in Imcivree™, an anorexigenic agent utilized in the treatment of obesity resulting from a rare genetic disorder. Or others, serving as drug-antibody linkers like Polatuzumab Vedotin-Piiq in Polivy™ listed in Table 1, linking monomethyl auristatin E (MMAE) with monoclonal antibody (mAb). Generally, FDA-approved therapeutic peptides are administered via 3 different routes: intravenous, oral, and subcutaneous. As indicated in Table 1, intravenous delivery stands out as the most chosen route in the design of peptide-based drugs.

**Table 1:** A list of peptide drugs approved by the US-FDA since 2018

Active ingredient (Brand)	Indication	Receptor	Administration route	Reference
[^177^Lu]Lu-DOTA-TATE ([[^177^Lu]Lu-DOTA^0^, Tyr^3^]-octreotate) (Lutathera^®^)	Gastroenteropancreatic neuroendocrine tumors (GEP-nets) in adults	Somatostatine receptor in tumor cells	Intravenous	[54]
Afamelanotide (Scenesse^®^)	Erythropoietic protoporphyria (EPP)	G-protein-coupled MC1R in dermal cells	Subcutaneous	[55]
Bremelanotide (VYLEESI™)	Hypoactive sexual desire (HSDD) in women of fertile age	Melanocortin receptors	Subcutaneous	[55]
Enfortumab Vedotin-Ejfv (PADCEV™)	Adult patients with locally advanced or metastatic urothelial cancer who previously received immune checkpoint therapy	Poliovirus receptor-related protein 4 (PVLR4)	Intravenous	[55]
Polatuzumab Vedotin-Piiq (Polivy™)	Adults with relapsed or refractory diffuse large B-cell lymphoma	Mature B-cells	Intravenous	[55]
Setmelanotide (Imcivree™)	Obesity caused by certain variants of the genes encoding for pro-opiomelanocortin (POMC), proprotein convertase subtilisin/kexin type 1 (PCSK1), or leptin receptor (LEPR)	Melanocortin-4 (MC4) receptor	Subcutaneous	[56]
Belantamab Mafodotin-Blmf (blenrep™)	Relapsed or refractory multiple myeloma in adults	BCMA cell	Intravenous	[56]
Vosoritide (voxzogo™)	Achondroplasia genetic disorder	Natriuretic peptide receptor B (NPR-B)	Subcutaneous	[57]
Melphalan Flufenamide (Pepaxto^®^)	Multiple myeloma (MM) and amyloid light-chain amyloidosis	Myeloma cells	Intravenous	[57]
Voclosporin (lupkynis™)	Lupus nephritis in adults	T-cell	Oral	[57]
Pegcetacoplan (empaveli™)	Paroxysmal nocturnal hemoglobinuria (PNH) in adults	Complement protein C3 and its activation fragment c3b	Subcutaneous	[57]
Dasiglucagon (zegalogue™)	Severe hypoglycemia in diabetic patients aged over six years	Glucagon receptor in hepatocytes	Subcutaneous	[57]
Difelikefalin (korsuva™)	Moderate-to-severe pruritus associated with chronic kidney disease (CKD-ap) in adults undergoing hemodialysis	Kappa opioid receptor in nerve cells	Intravenous	[57]
Odevixibat (Bylvay™)	Pruritus in patients over three months of age with progressive familial intrahepatic cholestasis (PFIC)	Ileal bile acid transporter (IBAT) in the distal ileum	Oral	[57]
Semaglutide (Rybelsus^®^)	Adults with type 2 diabetes mellitus	Glucagon-like peptide-1 (GLP-1) receptor	Oral	[58]

## Properties of pSi and MSN ideal for drug delivery

Porous materials have a long history of use as drug carriers. Among the various porous materials, pSi [59] and MSNs [37] are two of the most common drug carriers. Several successful attempts have been made in loading therapeutic cargos from small molecules (drugs) to macromolecules (proteins, nucleic acids) within the nanosized porous channels of pSi and MSNs for therapeutic delivery applications [60–62]. For instance, the first study utilising MSNs as carriers was published in 2001 by Vallet-Regi et al., which utilised MSNs to enhance the solubility of ibuprofen, a lipophilic drug [63]. Similarly, the first study incorporating drugs into pSi was published in 2004 by Anglin et al. [64]. This study loaded steroid dexamethasone into freshly etched pSi to achieve controllable drug leaching and dissolution [64]. Both pSi and MSNs have attracted significant attention for drug delivery due to their unique features such as biocompatibility, large pore size, uniform distribution, and extensive surface area [38, 65–67]. The following sections provide a brief overview of the unique properties that make pSi and MSNs an ideal drug carrier for a range of therapeutic payloads.

## Fabrication and key properties of pSi

Porous silicon (pSi) typically displays nanosized open pores that can serve as containers to incorporate therapeutic payloads effectively. The porous structural features drastically increase the surface areas, hence befitting a suitable system for loading and controlled elution of drug molecules. Most often, pSi materials are synthesised using an electrochemical etching-based “top-down” synthesis approach. In this method, silicon wafers are etched in the presence of hydrofluoric acid (HF) based electrolytes, where the silicon wafers serve as the anode against a Platinum cathode [38, 68]. The pores reach the hydrogen-terminated surface of silicon during the etching process, resulting in the nucleophilic attack of fluoride ions on the Si-H bond, resulting in the formation of Si-F bonds [38, 68]. The pore size of the pSi is controlled by varying etching parameters such as current density, concentration of electrolyte, crystal orientation of Si wafer, and concentration and type of dopants used. Some other methods used for pSi synthesis include photochemical, stain, gas-induced, and spark-induced etching [68]. The pSi layer attached to the underlying Si wafer can be easily removed to create free-standing porous films that are milled ultrasonically or mechanically to obtain micron- or nano-sized porous sheet-like particles. In the particle form, the pSi can be applied for both depot and systemic delivery of therapeutic payloads. Several studies have shown that the pore structure and surface chemistry of pSi contribute to improved drug adsorption, solubility, and drug release [38, 65, 69]. It is worth mentioning that even though native pSi particles remain stable in the air while being stocked, there is a tendency that they can be unstable while in contact with physiologically relevant solvents [70, 71]. Novel stabilisation chemistries have been developed over the last two decades to improve the biological stability of pSi to ensure stability during drug loading and handling process, while still retaining biodegradability potential. For instance, partial oxidation of pSi particles (TOPSi) by a thermal method using mild temperatures incorporates hydrophilic properties with moderate stability [72, 73], while the thermal hydrocarbonization process enables obtaining more stable pSi having a hydrophobic surface covered with hydrocarbons (THCPSi). On the other hand, novel chemical procedures incorporating the thermal functionalisation of THCPSi along with undecylenic acid (UnTHCPSi) have moderately hydrophilic surfaces having hydroxyl groups that can be further modified as per desirable functional groups [74]. These surface chemistries are key to both handling stability of pSi as well as optimal and precisely controlled high drug loading capacity of this material, which can be tuned according to the physiological properties of the drug molecule. In this regard, a study unveiling the pharmacokinetic properties of pSi and peptide YY3-36 (PYY3-36), an endogenous peptide belonging to the family of neuropeptide Y and pancreatic polypeptide (PP) showed the ability of pSi particles to load peptide along with its possible controlled release in both in vitro and in vivo system [75]. Moreover, TOPSi showed maximum release ability along with rapid degradation rate followed by THCPSi and UnTHCPSi in both in vitro and in vivo systems [75]. A study investigating the use of anionic TOPSi and cationic TOPSi for in vivo delivery of glucagon-like peptide -1 (GLP-1) reported better efficacy of cationic pSi [76]. In another study, loading of PYY3-36 onto pSi showed prevention of peptide degradation as well as its increased bioavailability [61, 77], hence suggesting that tailored controlled formulation of pSi could be effective means of delivery of peptide for therapeutic use. Additionally, methodological simplicity in the loading procedure ensures that even the most sensitive drug payloads like antibodies and peptides remain stable during preparation, while the pores protect the payload from harsh biological conditions during the administration [68, 75].

Moreover, pSi exhibits inherent photoluminescence (PL) in the red and near-infrared region of the spectra, which can be utilised for imaging and tracking of the particles. Sailor et al. first presented luminescent pSi nanoparticles (LPSiNPs) for biological applications. The inherent red-NIR range PL of LPSiNPs was utilised to track the biodistribution and accumulation of particles in vivo. They observed that the PL of the LPSiNPs visibly diminished within one week and completely cleared within four weeks. This clearance mechanism was attributed to their degradation into silicic acid, subsequently facilitating excretion from the body [70].

## Fabrication and key properties of MSNs

MSNs have emerged as an ideal drug carrier due to their unique properties and versatile applications in the field of drug delivery. MSNs possess a mesoporous structure with well-defined and uniform pores, making them capable of efficiently encapsulating and delivering a wide range of therapeutic agents [78]. Chemically, MSNs have a honeycomb-like structure and an active surface area that enables the functionalisation of particles, facilitating the incorporation of desirable surface properties and linking with specific molecules [67, 79]. This property is vital for their application in drug delivery systems. The synthesis of MSNs typically occurs at low surfactant concentrations, involving the interaction between anionic oligomers of orthosilicic acid and cationic surfactants, which alters the mesophase structure to a smaller size [80, 81]. One of the key advantages of MSNs is their high surface area, providing ample space for drug loading. The large surface area-to-volume ratio of MSNs allows for high drug loading capacity, enabling the delivery of a substantial amount of drugs in a compact carrier [82].

Moreover, the mesoporous nature of MSNs ensures controlled and sustained release of the encapsulated drugs, allowing for precise modulation of drug release kinetics [83]. The tunable pore size of MSNs is another crucial attribute contributing to their suitability as drug carriers [78]. By adjusting the synthesis parameters, such as surfactant concentration or template size, the pore size of MSNs can be tailored to accommodate different types of drugs, including small molecules, proteins, peptides, and nucleic acids. This versatility in pore size control enables efficient loading and release of a wide range of therapeutics [84]. The biocompatibility of MSNs further enhances their applicability as drug carriers. MSNs comprise silica, a biocompatible material well-tolerated by the human body. This biocompatibility minimises the potential for adverse reactions and ensures the safety of MSNs as drug-delivery vehicles [82].

The stability of MSNs is another important aspect that makes them an ideal drug carrier. The stability of MSNs is primarily attributed to the formation of Si-O bonds within the silica structure. Several core elements, such as tetraethyl orthosilicate (TEOS), tetramethoxyvinylsilane (TMVS), and tetramethyl orthosilicate (TMOS), serve as significant silica precursors in the synthesis of MSNs [85]. MSNs exhibit excellent thermal and chemical stability, ensuring the integrity and longevity of the encapsulated drugs during storage and transportation [86]. Moreover, the silica framework of MSNs protects the encapsulated drugs from degradation, enzymatic activity, and harsh physiological conditions. The versatility of MSNs extends beyond conventional drug delivery. They can also serve as multifunctional platforms for theranostic applications, combining therapeutic and diagnostic functionalities [87]. MSNs can be loaded with imaging agents, such as fluorescent dyes or contrast agents, enabling real-time monitoring of drug release, biodistribution, and therapeutic efficacy [88].

The surface of MSNs can be modified or functionalised with various molecules, such as targeting ligands or cell-penetrating peptides, to enhance their specificity and selectivity towards target cells or tissues [89]. In contrast to porous silicon (pSi), MSNs are synthesised using "bottom-up" approaches. The commonly employed methods include the solution-based method (SBM) and the Sol-Gel process. In SBM, alkyl ammonium salts, such as cetyl trimethyl ammonium bromide, undergo liquid crystallisation and concentration at the hydrophilic interface through electrostatic and hydrogen bonding interactions with silica precursors, leading to the formation of mesoporous products [66, 67]. The Sol-Gel process involves hydrolysis and condensation reactions to produce colloidal particles in an aqueous phase, with pH level adjustments to accelerate the process [90, 81]. The size of MSNs obtained through this process ranges from 2 to 1000 nm, and the choice of synthesis method depends on the desired characteristics and size of the particles [67, 90]. Another method for MSNs synthesis is evaporation-induced self-assembly, which necessitates the formation of homogeneous soluble silica solutions in ethanol and water, typically with a micelle concentration of surfactant [91, 92].

MSNs and pSi materials have unique physiological properties such as biocompatibility, systemic stability, pH resistance, and hydrophobicity. Both pSi and MSNs materials have been explored for targeted delivery of drugs along with application in controlled drug release, cell tracer biosensor or diagnostic tool [62, 93, 94]. Nanoparticles, specifically mesoporous silica particles (MSPs), have shown promise for delivering antimicrobial agents against intracellular infections like Mycobacterium tuberculosis. In this study, researchers discovered a peptide called NZX that effectively inhibits different strains of M. tuberculosis. They investigated the potential of MSPs loaded with NZX for tuberculosis treatment. The NZX-loaded MSPs released the peptide gradually in simulated lung fluid, and primary macrophages readily took up the particles. In an intracellular infection model, the NZX-loaded MSPs demonstrated superior efficacy in killing mycobacteria compared to free peptides. The therapeutic potential of the peptide-loaded MSPs was further validated in a murine infection model, where they successfully eliminated M. tuberculosis. These findings emphasise the enhanced inhibition of intracellular mycobacteria in primary macrophages and the capability of MSPs to eradicate *M. Tuberculosis* in vivo [95].

## Peptide delivery using pSi

Due to its superior loading capacity, biodegradability and biocompatibility [38], pSi shows great potential in drug delivery systems and has been successfully utilised as a carrier for peptides in the last two decades [24, 96]. Moreover, modifying the surface of pSi nanoparticles can enhance the effectiveness of drugs by enabling them to specifically bind to their corresponding receptors through conjugation [97], including carbohydrates [98], polymers [99], lipids [100], and antibodies [101].

**Fig. 2:**
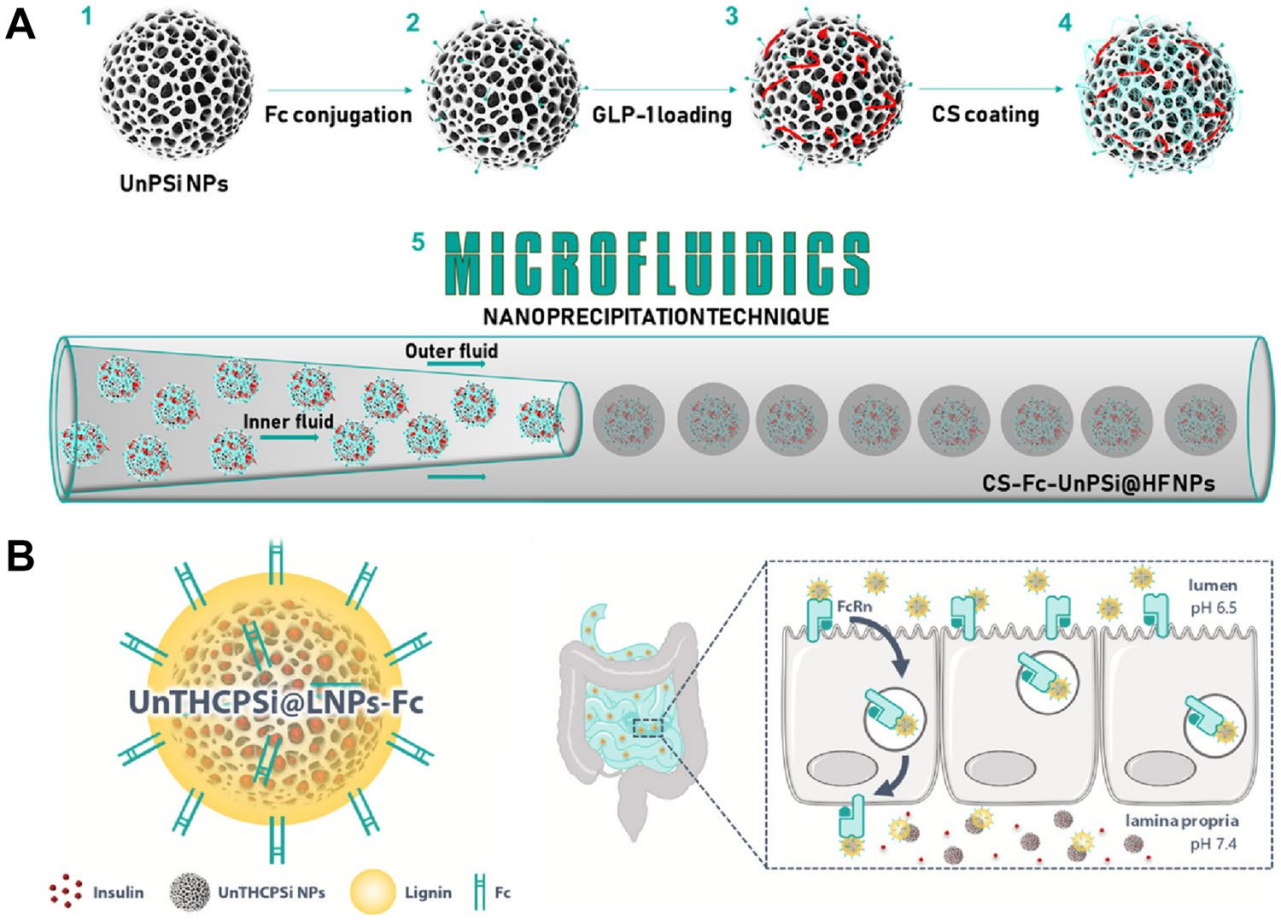
Surface modification of pSi nanocarriers for pH-responsive oral delivery. **A** Illustration of the fabrication process for Fc-UnPSi NPs loaded with GLP-1 and the setup of the microfluidic device utilized for nanoprecipitation. Adapted with permission from [109]. Copyright 2018 American Chemical Society. **B** Diagram of the nanostructure, featuring insulin-loaded porous silicon nanoparticles (NPs) enclosed within a pH-responsive lignin matrix (LNPs). The NPs are functionalized on the surface with the Fc moiety of IgG to enhance transport across intestinal cells through the FcRn-mediated transcytotic pathway. Retrieved from [110], Elsevier Bioactive Materials

Systemic administration of therapies allows for fast distribution and 100% bioavailability. Therefore, this is the most widely utilised route for the delivery of peptide-based therapeutics [102]. However, fast clearance and digestion of the peptides in the circulation have led the researchers to utilise the porous silicon as a carrier to overcome these issues [103, 104]. In this regard, Kovalainen et al*.* loaded a gut hormone, peptide YY3–36 (PYY3-36), in pSi nanoparticles to treat obesity. The study explored both intravenous (i.v.) and subcutaneous administration of PYY3-36 loaded pSi. The i.v. administered particles appeared to clear out from the circulation quickly, while the subcutaneously injected particles provided a sustained peptide dose for regulating energy homeostasis. The findings also suggest that altering the surface chemistry of the nanocarriers to form thermally hydrocarbonised pSi (THCPSi) can influence the release rate of the peptide, allowing for better control over the release characteristics [75]. Majority of efforts on utilising pSi as peptide carriers have focused on their oral delivery. Particularly, researchers have explored use of modified pSi nanoparticles for oral delivery of antidiabetic peptide drugs aiming to prevent their degradation in the digestive tract and improve their systemic bioavailability [105–107]. In this area, Santos’s lab has reported the use of pSi for oral delivery of insulin and glucagon-like peptide-1 (GLP-1) for the management of diabetes. In an in vitro study, their group formulated a multi-drug delivery system by engineering layered polymeric coating onto pSi particles. In this work, Chitosan was covalently linked to pSi particles to provide mucoadhesive features to the particles loaded with GLP-1. An enteric coating of hydroxypropyl methylcellulose acetate succinate (HPMCAS-MF) that incorporated dipeptidyl peptidase-4 (DPP4) inhibitor was crafted around the chitosan modified GLP-1 loaded pSi using an aerosol reactor technology [108]. The particles displayed a pH-dependent change in size that went from approximately 800 nm at pH 1.2 to around 200 nm at pH 6 and beyond. This is attributed to the rapid dissolution of the HPMCAC-based enteric layer at elevated pH values that is also related to the pH-dependent release of GLP-1, while the release of DPP4 only embedded in the outer enteric shell showed no difference in release with pH change. Martins et al. developed Fc-conjugated undecylenic acid-modified THCPSi nanoparticles (Fc-UnPSi NPs) for GLP-1 oral delivery. The particles were coated with mucoadhesive chitosan and incorporated into a pH-responsive HPMCAS matrix using microfluidic nanoprecipitation as shown in Fig. 2A. The resulting nanoparticles exhibited controlled size distribution and sustained release of the payload over a period of 6 h. The functionalisation of NPs with the Fc fragment enhanced cytocompatibility and increased interaction with intestinal cells. Furthermore, the Fc-conjugated NPs demonstrated improved Glucagon-like peptide-1 (GLP-1) permeability in an intestinal in vitro model [109]. Later in 2022, Martins et al. reported another nanosystem wherein insulin-loaded pSi was enveloped within a pH-responsive lignin matrix and surface-functionalized with the Fc fragment of immunoglobulin G as a targeting ligand for the neonatal Fc receptor (FcRn). The NPs demonstrated a small size (211 ± 1 nm) and remained intact in stomach and intestinal pH conditions, releasing the drug exclusively at pH 7.4, as shown in Fig. 2B. Cytocompatibility tests confirmed the formulation's safety, while plasmon resonance studies demonstrated enhanced interaction and internalisation of the FcRn-targeted NPs by Caco-2 cells expressing FcRn. Moreover, in vitro permeability studies conducted using a Caco-2/HT29-MTX co-culture model revealed that the Fc-functionalized NPs significantly augmented insulin permeation compared to non-functionalized NPs [110]. This oral-delivered insulin could potentially increase type 1 diabetes mellitus patient compliance without long-term repeated injection. In a different approach, inspired by the surface properties of viruses, Rao et al. developed a novel platform, namely poly (pyridyl disulphide ethylene phosphate)/sulfobetaine modified amine-modified pSi nanoparticles (P(PyEP-g-SB)-AmPSiNPs), for enhanced oral insulin delivery. The optimised formulation, P(PyEP-g-SB0.3) 20-AmPSiNPs, exhibited improved mucus penetration, enhanced cellular uptake, and enhanced epithelial permeability. The modified P(PyEP-g-SB0.3)20-AmPSiNPs showcased notable advantages in overcoming mucus barriers and facilitating cellular uptake, resulting in a significant improvement in the oral bioavailability of insulin (4.36%) and 2.08-fold higher than free insulin solution (2.09%). This study emphasises the potential of the P(PyEP-g-SB)-AmPSiNPs platform as an efficient oral delivery system, particularly for sensitive drugs such as proteins [111].

Apart from particle forms of pSi, devices made from silicon that display surface porosity can be classified as pSi material due to their porous nature and silicon-based chemical matrix. For example, two types of porosified silicon-based microneedle (MN) devices have been used to deliver peptide payloads transdermally [112]. Resnik et al. introduced an innovative in vivo insulin delivery approach via microinjection, utilising a hollow pSi MN array, and subsequently characterised the efficacy of drug transfer using methylene blue as a tracer. The research findings indicate that the transfer efficiency is largely constrained by the viable epidermis's limited capability to absorb and facilitate enhanced drug transport towards the capillary-rich region. The in vivo tests demonstrated successful infusion of rapidly acting insulin, a fact substantiated by blood analyses. When compared with subcutaneous delivery involving nearly equivalent infusion dosages, pSi MN delivery exhibited a comparatively lesser decline in glucose levels, accompanied by a noteworthy elevation in serum insulin (40–50%), which is primarily attributed to the more effective delivery of concentrated (200 IU/mL) exogenous insulin. In general, the designed pSi MN ensured the delivery of insulin while maintaining a relatively consistent plasma concentration. Notably, in terms of patient compliance, the pSi MN delivery method was found to be painless and devoid of skin irritation or inflammation at the delivery sites [112]. A number of studies have functionalised pSi with homing peptides to enhance the tissue-specific targeting of pSi. Although the incorporated peptide plays no therapeutic role, this indirect peptide delivery approach is popular in nanomedicine where tissue homing capabilities are paramount to avoid the side effects of the incorporated drug payload. For example, cysteine-alanine-glutamine-lysine (CAQK)-mediated siRNA delivery, the pSi NPs were functionalised with CAQK peptides on their surface. CAQK is a proteoglycan complex known to be upregulated in instances of brain injury. The CAQK peptides function as targeting ligands, designed to precisely recognise and bind to receptors or molecular markers expressed on cells at the intended location. This was particularly relevant for mice with acute brain injury, specifically targeting and interacting with the injured brain cells. The results demonstrate that CAQK-coated nanoparticles containing silencing oligonucleotides have translational potential because they can direct CAQK peptides to the site of acute brain injury after 5 days of systemic injection and retain it there for 3 h as a therapeutically relevant timescale. This approach offers an alternative to invasive local delivery methods, which can introduce complications to the injury site [113]. In another study, Sailor et al. devised a pSi-based nanoformulation using miR-21, a microRNA overexpressed in ovarian cancer, to inhibit tumour growth. This engineered nanoformulation utilised biodegradable pSi as a carrier, encapsulating an anti-miR-21 locked nucleic acid payload (with a loading efficiency of 17% by mass) and a tumor-homing peptide CGKRK for precise targeting. Following administration, the Quasar 670-labelled anti-miR-21 CGKRK–pSi exhibited fluorescence intensity threefold higher in tumour cells compared to controls, thus affirming the effective tumour-homing potential of the CGKRK targeting peptide. Upon administering anti-miR-21 CGKRK–pSi to mice, a complete suppression of tumour growth was achieved, with no discernible increase in total tumour volume even after a period of 10 days. In stark contrast, control groups treated with PBS or CGKRK–pSiNPs containing scrambled LNA demonstrated a substantial tenfold increase in tumour growth. This outcome unequivocally validates the significant inhibition of tumour growth accomplished through the targeted delivery of miR-21 silencing facilitated by pSi [114].

## Peptide delivery using MSNs

MSNs have a number of unique features that make them attractive for drug delivery, including their high surface area, tunable pore size, and biocompatibility. The tuneable porous nature of MSN is particularly attractive for loading and delivery of biological therapeutics [78]. In the context of peptide delivery, MSNs can be synthesised with tailored pore sizes, surface chemistries and functionalities, providing a versatile platform for encapsulation, protection, and delivery of peptides [115]. MSNs can similarly improve the pharmacokinetics of peptides by prolonging their circulation half-life. The properties of MSNs enable a sustained and localised release of peptides, leading to improved therapeutic outcomes [116]. MSNs can be tuned by adding different functional groups to their surface, improving their ability to carry and deliver peptides to specific cells or tissues. For example, positively charged surface modifications can electrostatically interact with negatively charged peptides, promoting their encapsulation. Beyond encapsulating peptides within MSNs, applying a polyethylene glycol (PEG) coating to nanoparticles can enhance their biocompatibility and reduce the likelihood of immune system recognition[117]. The design of MSNs-based peptide delivery systems also allows for multifunctionality. In the context of peptide delivery, MSNs can be synthesised with tailored pore sizes, surface chemistries, and functionalities, providing a versatile platform for encapsulation, protection, and delivery of peptides [115]. The properties of MSNs enable a sustained and localised release of peptides, leading to improved therapeutic outcomes [116]. Using MSNs to deliver peptides is a versatile approach that allows for the simultaneous delivery of multiple types of therapeutic molecules to tumor cells at the subcellular level [118]. Attaching targeting ligands or antibodies to the surface of MSNs allows them to specifically recognize and bind to target cells or tissues, which can improve the effectiveness of the treatment and reduce side effects [119–121].

**Fig. 3:**
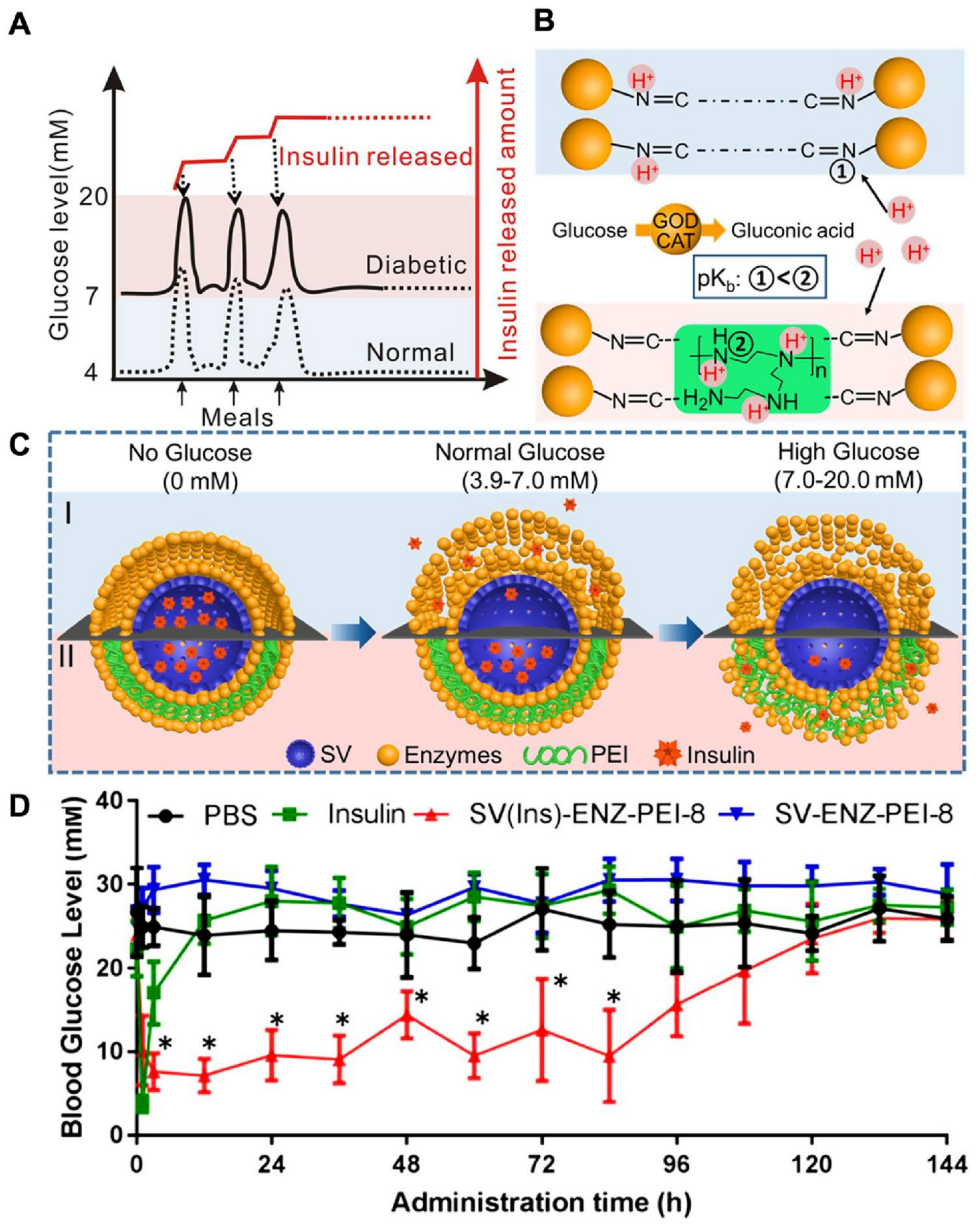
A schematic illustration of glucose-responsive insulin delivery systems based on MSNs. **A** Optimal insulin-release profile across different blood glucose levels. **B** The mechanism of insulin release from glucose enzyme–PEI systems. **C** Contrasting conventional and physiological glucose-responsive insulin-release mechanisms. **D** In vivo results demonstrating swift insulin release in response to glucose. Adapted with permission from [122]. Copyright 2019 American Chemical Society

Utilising the multifunctional features of MSNs, silica vesicle-based glucose-responsive insulin delivery system with an enzymatic polymer layer-by-layer (LBL) coating was presented by Xu et al*.* For the first time, they reported that the insulin-release thresholds could be changed to a desirable glucose concentration. The researchers used a polyethyleneimine that can bind to protons and glucose-specific enzymes to coat the MSNs with a concentration range of 5–20 mM. Furthermore, in vitro studies, they found that the nanosystem consistently released insulin in response to high glucose levels (10, 20 mM) and stopped releasing insulin in response to normal glucose concentrations (5 mM). In experiments with mice with type 1 diabetes, the nanosystem rapidly released insulin and maintained normal blood sugar levels for up to 84 h with a single injection, without affecting the blood sugar levels of healthy mice (Fig. 3) [122]. MSNs are widely used in immunotherapy and vaccine development for efficient peptide delivery. They are effective carriers for peptide antigens, facilitating precise and targeted immune responses. In vaccine development, MSNs protect peptide antigens, enhancing their stability and immunogenicity. The versatility of MSNs enables the incorporation of multiple antigens, enabling the development of multi-epitope vaccines. The researchers introduce a simple yet effective technique for producing uniform and stable hollow mesoporous silica nanoparticles (HMSNs). These HMSNs are tailored to carry two peptides derived from melanoma, namely HGP10025–33 and TRP2180–188, each possessing different levels of hydrophobicity. The peptides loaded HMSNs (referred to as HT@HMSNs) are enclosed within a lipid bilayer that contains the adjuvant monophosphoryl lipid A (resulting in HTM@HMLBs). This process enhances the overall stability, compatibility with biological systems, and efficiency of delivering multiple elements, including the peptides and the adjuvant. These HTM@HMLBs exhibit improved uptake by dendritic cells (DCs), leading to a more effective maturation process for these cells. Consequently, this maturation prompts the activation of T lymphocytes that are specific to the tumor, including both CD8+ and CD4+ types [123]. In another insulin delivery work, Zhao et al*.* [124] developed a new insulin delivery system that uses glucose oxidase (GOD). They used mesoporous silica nanoparticles (MSNs) with large pores to carry the insulin. They coated the MSNs with a layer of GOD and catalase (CAT) to seal the pores and control the release of insulin. The GOD and CAT layers were attached to the MSNs using a technique called layer-by-layer (LBL) assembly. The glucose-sensitive enzymatic layers were formed through Schiff base bond formation, allowing for insulin release in the presence of glucose. When glucose is present, the GOD and CAT enzymes react and release insulin from the MSNs [124]. MCM-41 type MSNs that exhibit unidirectional and highly uniform cylindrical porous channels with a hexagonal arrangement. It was utilised for the delivery of (KLAKLAK)2, an antibiotic peptide with disulphide linkages. Then followed by coating with charged reversal polyanion poly(ethylene glycol)-blocked2-3-dimethyl maleic anhydride-modified poly(L-lysine) (PGG-PLL(DMA)) through electrostatic interactions [66].

The researchers successfully loaded exenatide (EXT), a 39-amino acid peptide, into mesoporous silica nanoparticles (MSNs) of the SBA-15 type, which have hexagonally arranged unidirectional pores like MCM-41 (Fig. 3). Due to their large surface area, SBA-15 particles had a remarkable loading capacity of up to 15% w/w for EXT. These nanoparticles proved to be effective carriers for EXT, enabling the sustained release of the peptide in both in vitro and in vivo pharmacokinetic studies. The use of SBA-15 as a drug carrier extends the circulation time and significantly improves the bioavailability of EXT. Notably, EXT-SBA-15 has a longer hypoglycemic effect than using the EXT solution alone [125]. Scientists developed a new drug delivery system using amino-functionalized dual-mesoporous silica nanoparticles (N-EDMSNs) to deliver liraglutide and fibroblast growth factor 21 (FGF-21) simultaneously. The N-EDMSNs exhibited high gene-loading capacity and low toxicity when tested on Hepa1-6 cells. Additionally, the N-EDMSNs successfully delivered FGF-21 plasmids and liraglutide into Hepa1-6 cells. In mouse, treatment with N-EDMSNs carrying pFGF21 increased FGF-21 expression in the liver more than hydrodynamic delivery of pFGF21 alone. N-EDMSNs carrying both pFGF21 and liraglutide reduced blood glucose levels, body weight, and food intake significantly more than separate treatments involving pFGF21 and liraglutide. N-EDMSNs also increased energy expenditure and improved hepatic insulin resistance (IR) in mice on a high-fat diet (HFD) [126].

**Fig. 4:**
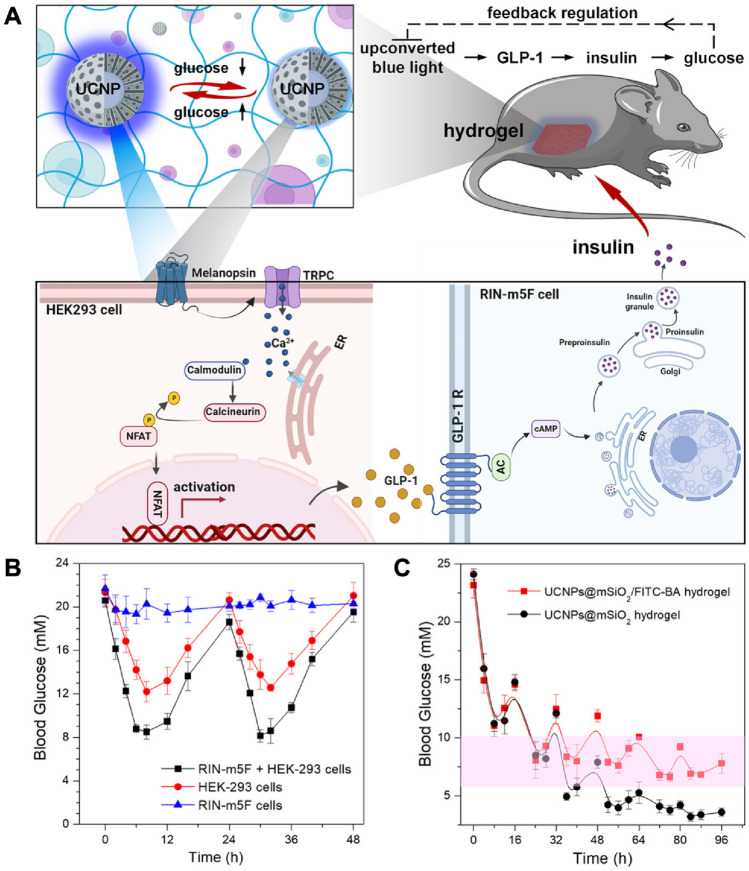
Regulating insulin secretion through a MSNs-based hydrogel system. **A** Diagrams illustrating the composite hydrogel for glycemic regulation in mice with diabetes. **B** Fluctuations in blood glucose levels in diabetic mice following the administration of hydrogels containing various cell line encapsulations. **C** Blood glucose fluctuations in diabetic mice following two distinct hydrogel treatments. Adapted with permission from [127]. Copyright 2023 American Chemical Society

Researchers developed a new way to deliver glucagon-like peptide-1 (GLP-1) and insulin to cells. They embedded silica-coated upconversion nanoparticles in a hydrogel, which encapsulates cells and allows light to trigger the release of the two hormones. The hydrogel consists of a mesoporous silica layer with upconversion nanoprobes that have fluorescein molecules modified with benzoboric acid (UCNP@mSiO2/FITC-BA). To create a reusable and reversible glucose detection method, they coated NaYF4:Yb/Tm nanoparticles with a mesoporous silica layer of the appropriate size. The FITC-BA molecule is hydrophobic because of its core component, fluorescein isothiocyanate. However, when exposed to higher glucose concentrations, it transitions into the hydrophilic FITC-Glu. Synthetic biology in conjunction with optogenetics holds the cell-based therapies in future. However, achieving precise control over genetic expression without reversible too.ls for real-time metabolite monitoring presents challenges. To tackle this issue, researchers have innovated a smart hydrogel platform that leverages analyte-induced hydrophobicity modulation within mesoporous silica. This platform integrates optogenetically modified cells with reversible upconversion nanoprobes responsive to glucose. The modulation of optogenetic activity, crucial for insulin secretion, is achieved by dynamically adjusting the intensity of blue light based on blood glucose levels. By employing near-infrared illumination, the hydrogel system maintains glucose balance, averting the risk of hypoglycemia [127], as shown in Fig. 4.

**Fig. 5:**
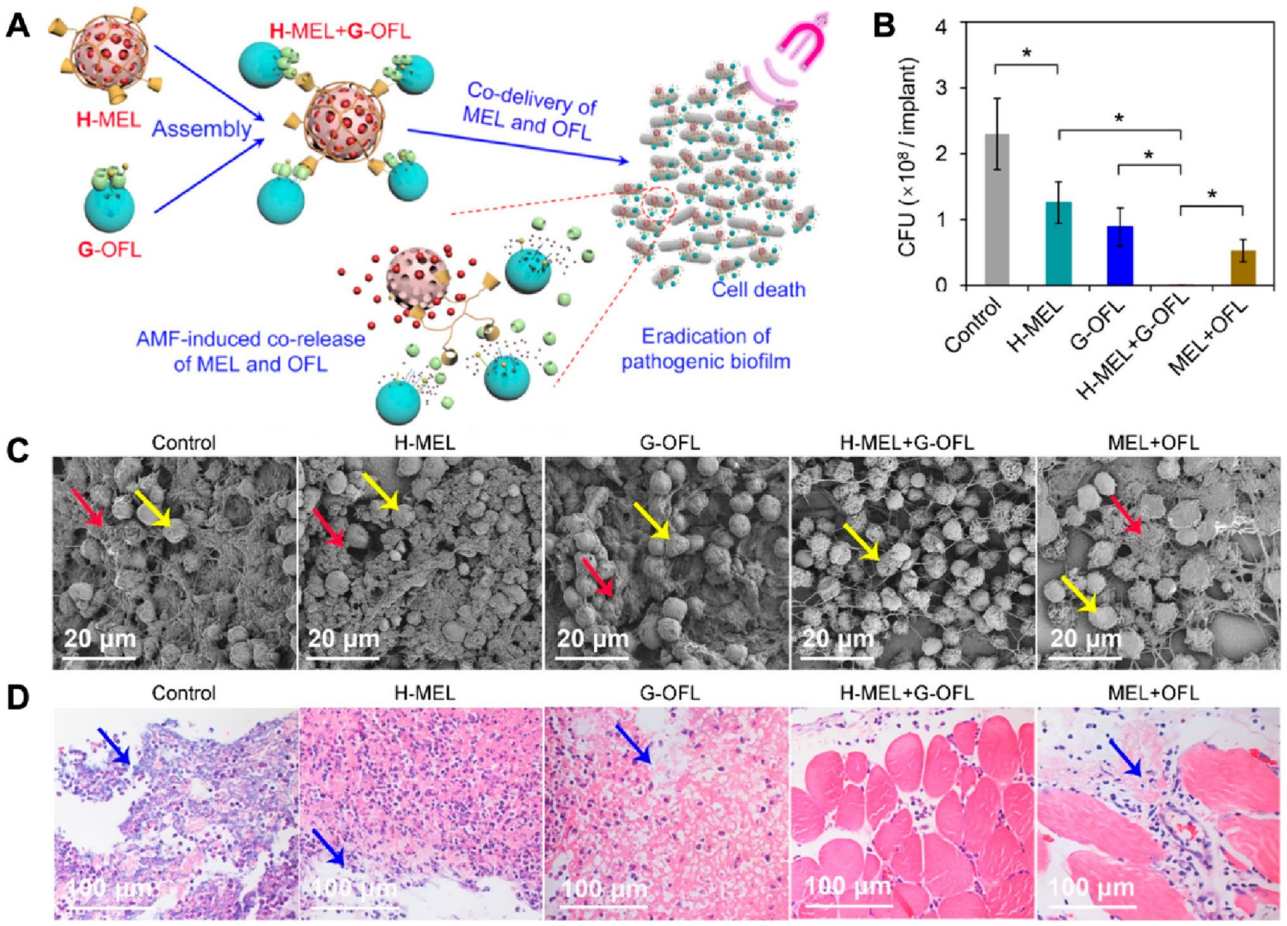
Antibacterial dual system for the delivery of pharmaceuticals and genetic material. **A** Diagrammatic representation of the molecular constituents and synthesis pathway of parent MSN (H), guest MSN (G), and coassemblies featuring H loaded with MEL (H-MEL) and G loaded with OFL (G-OFL). **B** Enumeration of microbial cells in biofilm-affixed implants. **C** SEM images of the internal surfaces of implants. Red arrows highlight representative bacterial cells within biofilms, while yellow arrows indicate the host cells. **D** Histopathological visuals of tissues at the implantation sites. Blue arrows point to compromised host muscle tissues experiencing inflammation. Adapted with permission from [131]. Copyright 2020 American Chemical Society

In groundbreaking work, scientists have developed a new way to deliver antimicrobial peptides to tumors using light-responsive mesoporous silica nanoparticles (MSNs). They loaded the peptide PA-C1b into MSNs and labelled them with a dye called sulfo-cyanine7 (Cy7). Then, they coated the MSNs with graphene oxide (GO) and attached folic acid to them so that they would specifically target tumors. This system allows to control the release of the peptide with light, which enables precise cancer therapy. The Cy7 dye helps them track the nanoparticles in real time, and the GO coating prevents the peptide from leaking out before it is exposed to light. When they irradiate the nanoparticles with light, the GO coating comes off, and the peptide is released. This system worked well in both cell experiments and in animal experiments with mouse tumors. It could be used to deliver antimicrobial peptides to treat infections and cancers, and it could potentially make peptide-based treatments more effective and specific [128]. Scientists have developed a new drug delivery system that uses mesoporous silica nanoparticles (MSNs) and a peptide-based amphiphile called ADDA-TCPP to deliver drugs to tumors in a controlled manner. This amphiphile has both hydrophobic and hydrophilic segments connected by a disulfide bond. ADDA-TCPP acts as a gatekeeper, sealing the MSN pores and preventing the drug from leaking out until it is exposed to a high concentration of glutathione, which is present at high levels in the cytoplasm of cancer cells. The system also includes an RGDS peptide, which helps it target tumor cells that overexpress the αvβ3 integrin receptor. This multifunctional nanosystem could be used to deliver a variety of drugs to cancer cells, and it has the potential to improve the effectiveness and safety of cancer treatment. Tat48-60 modification on MSNs enhanced intracellular drug delivery and exhibited significant toxicity against tumour cells. Tat48-60 modification on MSNs enhanced intracellular drug delivery and showed significant toxicity against tumour cells [129]. The goal of this research is to develop a biocompatible nanocarrier that can deliver both genes and drugs for advanced cancer therapy. This nanoplatform designed by Rong et al. (Fig. 5), called UCNPs(BTZ)@mSiO2-H2A, combines mesoporous silica nanoparticles with the anti-cancer drug bortezomib (BTZ). Adding H2A to the nanoparticles makes them more biocompatible and helps them encapsulate genes more effectively. The UCNPs(BTZ)@mSiO2-H2A/p53 nanoplatforms can effectively induce apoptosis in cancer cells that lack the p53 gene by co-delivering BTZ and restoring normal p53 function. Additionally, it enhances the sensitivity of p53-deficient non-small cell lung cancer cells to BTZ. The core-shell structured UCNPs@mSiO2 nanoparticles enable real-time monitoring of nanoparticle-cell interactions. The nanoplatform showed potential for clinical applications with its sustained release properties, upconversion luminescence imaging capability, and improved drug delivery potential [130].

MSNs, especially those with large pores, are promising for improving the oral delivery of vancomycin (Van) and other antimicrobial peptides [95, 131–133]. Van is a crucial antibiotic for treating systemic methicillin-resistant Staphylococcus aureus (MRSA) infections, but its poor membrane permeability necessitates costly intravenous administration. Researchers developed SNPs with different pore sizes and modified their surface properties using functional groups. Vancomycin-loaded SNPs exhibited controlled release compared to unencapsulated Van. Notably, Van-loaded SNPs, especially those with large pores and a negative charge, significantly enhanced Van's ability to cross an epithelial cell monolayer. This improvement can be attributed to the nanoparticles' ability to transiently open tight junctions, as evidenced by a decrease in transepithelial resistance (TER). Importantly, this effect was reversible within three hours. The development of MSNs for oral vancomycin delivery could revolutionize the treatment of MRSA and other serious infections [134].

## Conclusion and future opportunities

In theory, both pSi [61] and MSNs [135] offer comparable structural (i.e. particle size, pore diameter, surface area, and porosity), peptide loading capacity, and in-vitro and in-vivo biocompatibility. The pSi particles offer a greater tunability of pore size (2–200 nm) and porosity (up to 85%) their morphology is often sheet-like due to the top-down fabrication, which involves etching of a porous layer on a silicon wafer followed by lift-off and milling. The size of pSi particles can range from tens of nanometers to hundreds micrometers, whereas synthesis of micron-sized MSNs is challenging. In addition, MSNs, on the other hand, are typically spherical in shape and offer a narrow pore diameter tuneability but can achieve a surface area exceeding 1000 m^2^/g. All of these features have a huge impact on their peptide loading capacity. Peptide loading capacity has a direct impact on the efficaicy of the overall therapy, and therefore,an important factor to be carefully considered for selecting appropriate carrier [136]. In terms of loading capacity, both the carriers offer significantly higher peptide loading than the traditional lipid and polymeric carriers. For example, Shrestha et al*.* synthesised undecylenic acid-modified thermally hydrocarbonized pSi particles (171 ± 6 nm) with cell-penetrating peptide surface modification (CPP-CSUn, 258 ± 27 nm), achieving 17% average insulin loading capacity and 67% encapsulation efficiency [137]. Other studies have reported comparable loading capacity for pSi for various other peptides. A similar, close to 20 wt % peptide loading capacity has been reported for MSNs with many peptides. However, a study from Qin et al*.* utilised dendritic MSNs (165.4 ± 2.7 nm) with chitosan-g-3-fluoro-4-carboxyphenylboronic acid coating (CS-g-FPBA@MSN, 257.1 ± 4.4 nm), which showcased an extremely high insulin loading capacity of 29.5 ± 0.9% with encapsulation efficiency of 63.86 ± 1.7% [138]. These works show the ultrahigh peptide loading capabilities of both pSi and MSNs, while pointing out that pore morphology and surface chemistries of the particle play a crucial role in determining the final loading capacity [136, 139–143]. In addition to physical morphology and peptide loading capacity, biodistribution is another key factor that determines the disease site targeting success of a peptide loaded particle. In this regard, both pSi and MSNs display surface silanol groups which can be used to link targeting moieties to home the particles specifically to the disease site. Numerous studies have showcased this ability of pSi and MSNs successfully. However, the targeting ability and selectivity of the particles is predominantly dependent on the grafted targeting moiety (i.e. antibody, peptide), thus an objective comparison of the two particles is not justified.

**Fig. 6:**
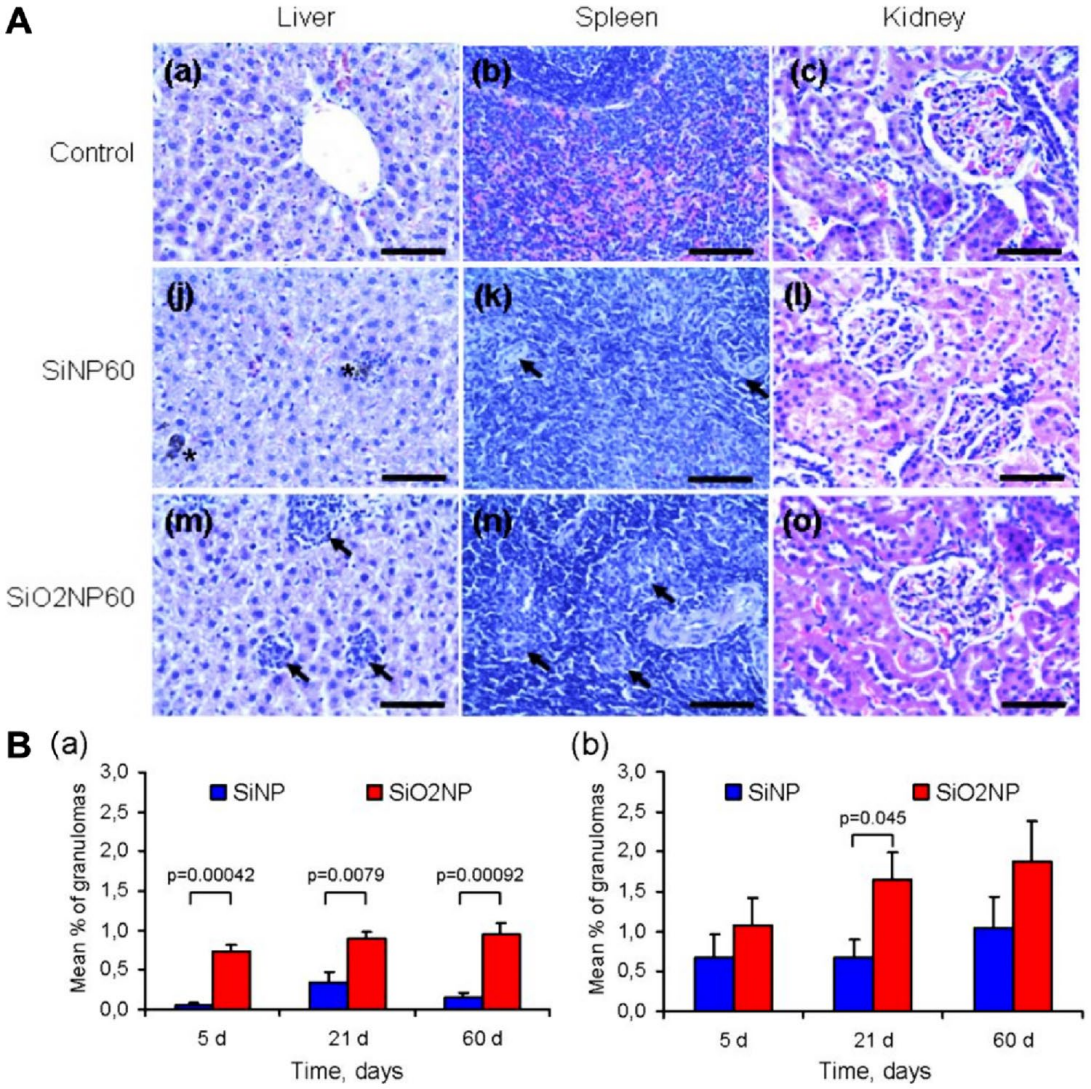
Long-term toxicity assessment of pSi and MSNs. **A** Histological examination of pSi and MSNs (SiNPs and SiO_2_NPs, respectively) 60 days after intravenous administration at a 7 mg/kg dose. The samples were fixed using paraformaldehyde and subjected to hematoxylin and eosin staining to assess any abnormalities. The scale bar represents 25 μm. **B** The average proportion of granulomas observed in the **a** hepatic and **b** splenic tissues at three distinct time intervals—5, 21, and 60 days following intravenous infusion. Retrieved from [144], MDPI

In-vitro and in-vivo biocompatibility is crucial to determine the suitability of a drug carrier for their clinical use. A vast number of studies have demonstrated the in-vitro and in-vivo biocompatibility of peptide loaded pSi and MSNs in a range of cellular and animal models. Notably, Shahbazi et al*.* discover that the toxicity response of immune cells (Raji, B-cell; Jurkat, T-cell; U937, monocyte; RAW 264.7 cells, macrophage) to five types of pSi nanoparticles (TOPSi, TCPSi, APSTCPSi, THCPSi, and UnTHCPSi) varies depending on the method of functionalisation. Their analysis revealed that the functionalisation of pSi nanoparticles resulted in different effects, such as DNA damage, changes in reactive oxygen species (ROS) and reactive nitrogen oxide species (RNOS), production of tumour necrosis factor-alpha (TNF-α), and levels of adenosine triphosphate (ATP), ultimately leading to apoptosis and mild organ inflammation in cells. The results also demonstrate that surface charge and hydrophilicity/hydrophobicity properties mainly contribute to cell cytotoxicity, while hemocompatibility was affected by two additional factors: exposure time and concentration. Thus, Shahbazi et al*.* pointed out that setting a dose and circulation time threshold was feasible to limit intravenous cytotoxicity [145]. Ferreira et al*.* found that a concentration of 50 μg/mL is the threshold for maintaining good cytocompatibility in cardiomyocytes for natriuretic peptide (ANP) modified undecylenic acid thermally hydrocarbonized pSi chelate with 1, 4, 7, 10-tetraazacyclododecanetetraacetic acid templates (Un-D-ANP) [146]. It is noteworthy that both Shahbazi et al*.* and Ferreira et al*.* have limited their in vivo investigations to a 24-h time point without continued tracking, which leaves a gap for long-term toxicity studies. To determine the relative long-term toxicity of MSNs, a total of 18 periodic intravenous injections were administered to 6 mice over 2 months. Except for one mouse that displayed mild, chronic, and multifocal gastritis, no histopathological abnormalities or treatment-related lesions were observed in the rest. Therefore, both conclusions reveal outstanding tolerance of MSNs [147]. A parallel in vivo toxicity study of pSi and MSNs found that neither the weight nor the inner ear function of tested rats was affected over 60 days of examination. However, importantly, both nanoparticles can induce granuloma formation in the liver and spleen, to which pSi has a lesser extent as compared to MSNs (Fig. 6A and B) [144]. It is noteworthy that while there may be a relative lack of comparative studies between pSi and MSNs in peptide delivery systems, this does not necessarily mean that one particle is superior to the other. Both pSi and MSNs have shown great potential in peptide/drug delivery applications, and the choice between the two may depend on various factors such as the specific peptide being delivered, the desired release kinetics, and the targeted tissue or organ. Additionally, it is important to consider the biocompatibility and toxicity of the particles, as well as their clearance mechanisms from the body. Therefore, further research is needed to fully understand the similarities and differences between these two types of nanocarriers and their respective advantages and disadvantages in peptide delivery applications.

Currently, numerous silica-based clinical trial studies are ongoing [148], and some silicon-based ones are also in progress [149, 150]. However, overall, clinical trials involving nanoparticles with peptide involvement are scarce, and those utilising peptides as therapeutic agents are even rarer. In 2014, Phillips et al. introduced Cornell dots, comprising a silica shell encapsulating the fluorescent Cy5 dye, coated with poly(ethylene glycol) (PEG), and functionalised with the integrin-targeting, radiolabeled peptide 124I-cRGDY. This groundbreaking study marked the first successful translation from animal to human subjects. Among five metastatic melanoma patients tested, PET imaging in two patients indicated the specific accumulation of Cornell dots in tumour regions. Metabolic studies revealed complete renal and bladder excretion of the particles with no observed toxicity. Phillips et al. advocate for subsequent research involving larger sample sizes in follow-up clinical trials [151]. In 2021, Zanoni et al. conducted a clinical study (NCT02106598) aimed at the real-time image-guided detection, localization, and surgical treatment of sentinel lymph nodes (SLN) in patients with head and neck melanoma. In 24 surgeries, 40 sentinel lymph nodes were excised. Preoperative localization of SLN was performed using technetium Tc 99 m sulphur colloid, followed by a particle dose escalation study, resulting in optimized doses and volumes of 2 nmol and 0.4 mL, respectively, with a maximum SLN signal-to-background ratio of 40. No adverse events were observed. Consistency in the assessment of SLNs, using technetium Tc 99 m sulphur colloid and cRGDY-PEG-Cy5.5-nanoparticles, was 90% (95% CI, 74%-98%), with five identified as metastatic. Ultra-bright nanoparticle fluorescence enables high-sensitivity SLN visualization, deep tissue imaging, and, in certain situations, detection through intact skin, aiding intraoperative identification without the need for extensive dissection of adjacent normal tissue or nerves. Results demonstrate that fluorescence-guided sentinel lymph node biopsy based on nanoparticles is feasible and safe in head and neck melanoma. Zanoni et al. propose that this technique holds promise for improving lymphatic mapping and sentinel lymph node biopsy procedures, potentially reducing procedural risks [152]. On the other hand, no pSi-peptide systems undergoing clinical trials were found. Therefore, it is currently imperative to advance further clinical trial investigations on peptide delivery systems that have demonstrated efficacy and safety in animal experiments, with the aim of assessing their suitability for widespread market application.

The comparative analysis of pSi and MSNs in the delivery of peptide therapeutics reveals distinct advantages inherent to each material. The pSi is notable for its broader pore dimension tuneability (up to several 100 nm), biodegradability and the ability to modify its surface for targeted delivery, making it particularly useful for large peptides and proteins. It must be noted that the fabrication process requires highly corrosive acid like hydrofluoric acid (HF) and preparation of uniformly sized pSi particles sized pSi particles requires extensive post processing and washing steps. MSNs, on the other hand, offer a highly uniform particle size with limited pore size range (less than 20 nm), which is sufficient for a broad range of peptides and smaller protein payloads. Due to the scarcity of comparative studies conducted under identical conditions for both types of particles, a direct comparison to determine superiority is not feasible. Therefore, selection between these two carriers is highly dependent on target peptide, route of administration, and target disease. Overall, the pore size must be carefully controlled to balance efficient loading with sustained release, avoiding premature leakage or degradation of peptides.

To summarise, this review has assessed the function of pSi and MSNs in peptide delivery via different routes of administration. Overall, pSi and MSNs share similar chemical and mechanical properties, making them suitable candidates for shielding peptide drugs from degradation during processing, storage, and administration. Based on current studies, both nanocarriers exhibit comparable yet noticeable effectiveness in terms of delivery efficiency and biocompatibility. However, more studies should be conducted to further evaluate the long-term in vivo toxicity. Direct comparative analysis between pSi and MSNs is indeed limited in the literature. Researchers have primarily focused on exploring the individual characteristics and applications of these materials rather than conducting head-to-head comparisons. Nonetheless, ongoing research efforts are gradually increasing our knowledge and understanding of pSi and MSNs for the delivery of peptides and other macromolecular therapeutics.

## Data Availability

N/A.
